# The batched stepped wedge design: A design robust to delays in cluster recruitment

**DOI:** 10.1002/sim.9438

**Published:** 2022-05-21

**Authors:** Jessica Kasza, Rhys Bowden, Richard Hooper, Andrew B. Forbes

**Affiliations:** ^1^ School of Public Health and Preventive Medicine Monash University Melbourne Victoria Australia; ^2^ Centre for Primary Care and Public Health Queen Mary University of London London UK

**Keywords:** cluster randomized trial, intracluster correlation, sample size calculation, within‐cluster correlation structure

## Abstract

Stepped wedge designs are an increasingly popular variant of longitudinal cluster randomized trial designs, and roll out interventions across clusters in a randomized, but step‐wise fashion. In the standard stepped wedge design, assumptions regarding the effect of time on outcomes may require that all clusters start and end trial participation at the same time. This would require ethics approvals and data collection procedures to be in place in all clusters before a stepped wedge trial can start in any cluster. Hence, although stepped wedge designs are useful for testing the impacts of many cluster‐based interventions on outcomes, there can be lengthy delays before a trial can commence. In this article, we introduce “batched” stepped wedge designs. Batched stepped wedge designs allow clusters to commence the study in batches, instead of all at once, allowing for staggered cluster recruitment. Like the stepped wedge, the batched stepped wedge rolls out the intervention to all clusters in a randomized and step‐wise fashion: a series of self‐contained stepped wedge designs. Provided that separate period effects are included for each batch, software for standard stepped wedge sample size calculations can be used. With this time parameterization, in many situations including when linear models are assumed, sample size calculations reduce to the setting of a single stepped wedge design with multiple clusters per sequence. In these situations, sample size calculations will not depend on the delays between the commencement of batches. Hence, the power of batched stepped wedge designs is robust to unexpected delays between batches.

## 1 INTRODUCTION

**FIGURE 1:**
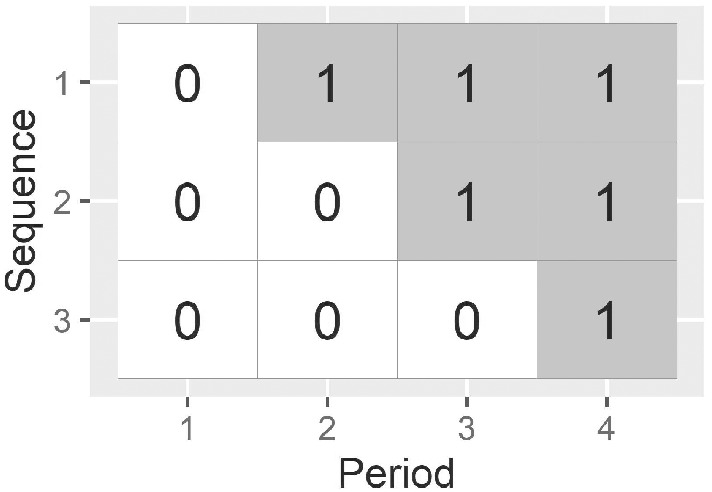
An example of a standard stepped wedge design, with 4 periods and 3 sequences (0 indicates periods in which the control condition is implemented; 1 indicates periods in which the intervention is implemented)

The stepped wedge cluster randomized trial design, where clusters are randomized to switch from a control to an intervention condition at different prespecified time points, has found application in a wide variety of research areas (examples in Mdege et al^1^). Figure 1 displays an example of a conventional stepped wedge design with four periods and three treatment sequences. The period lengths are typically of equal duration and define the times at which different clusters cross from the control to the intervention condition. Stepped wedge designs are useful when intervention conditions applied at the level of the cluster cannot be removed once implemented, for example, educational interventions, or when assessing changes in policy that will be rolled out across systems. A crucial advantage of stepped wedge trials is that they may require fewer clusters and smaller total sample sizes than standard cluster randomized trials, due to the within‐cluster comparisons enabled by the stepped wedge design.^2^ It is well‐recognized that the grouping of participants in clusters must be accounted for in sample size calculations and analysis of data from stepped wedge trials. In addition, due to the dependence between time and treatment in the stepped wedge, it is also essential to account for time in these calculations and analyses.^3^

**FIGURE 2:**
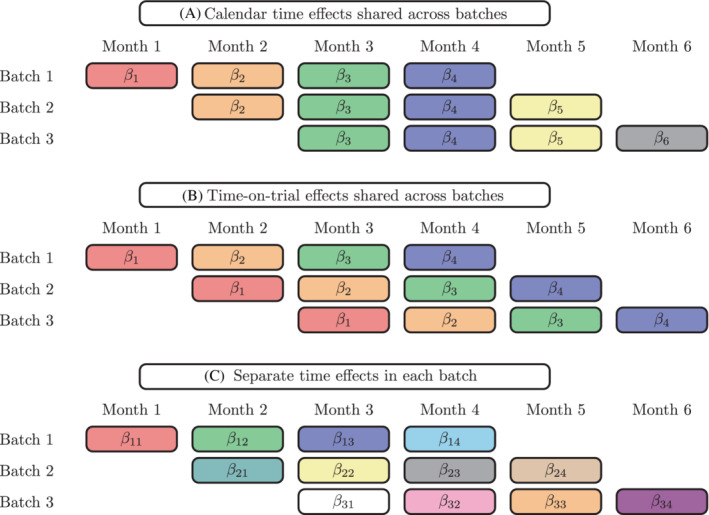
Three different ways in which the effect of time can be parameterized in a design where clusters commence study participation in three batches: the βs parameterize the time effects. For example, in the top panel, $\beta_{1}$ parameterizes the effect of month 1 on outcomes. The top panel indicates how effects of time are shared across batches when the effects of calendar time are assumed to be constant across batches; the middle panel indicates how the effects of time are shared across batches when time‐on‐trial effects are assumed to be constant across batches; the bottom panel indicates that no time effects are shared across batches when separate time effects are estimated in each batch

In the most commonly used sample size formulas and statistical models for the design and analysis of stepped wedge trials (eg, References 4, 5, 6) it is assumed that the effect of “time” on outcomes is identical across clusters, and that time is divided up into distinct trial periods. If clusters commence study participation at different times (ie, not all on the same date), then a distinction must be drawn between “calendar time” and “time‐on‐trial” (the amount of time since a cluster commenced trial participation). This distinction is particularly important for stepped wedge designs, where time and treatment are confounded. When clusters commence participation in a trial at the same calendar time, then calendar time and time‐on‐trial will be aligned: this is the case in Figure 1, where all clusters commence the study at the same time. When clusters are not aligned in calendar time, researchers must consider the distinction between calendar time and time‐on trial, and parameterize time to align with their assumptions about the effect of time on outcomes in their statistical models. Three different time parameterizations that could be chosen when clusters are not aligned in calendar time are displayed in Figure 2: calendar time effects could be shared across clusters; time‐on‐trial effects could be shared across clusters; or separate period effects could be assumed in each batch. If clusters are not aligned in calendar time, but a standard stepped wedge sample size formula is applied, the assumption is that time‐on‐trial has an identical impact across all clusters, and there is no impact of calendar time (corresponding to time‐on‐trial effects that are shared across batches as in the middle panel of Figure 2). Further, when the standard sample size formulas are applied, it is required that there are no systematic differences between the batches of clusters that commence trial participation at different time points.

In practice, the great majority of cluster randomized stepped wedge trials have been designed so that all the clusters commence their participation at the same calendar time. This is likely to be for two reasons: first because clusters may all have expressed an interest in collaborating from an early stage in the development of the trial, and are all ready to go when the trial begins, but second, perhaps, because of concerns about the most appropriate way to model calendar time vs time‐on‐trial, and the lack of methodological guidance. There may be situations where it is to a trialist's advantage to stagger the commencement of different clusters.

**FIGURE 3:**
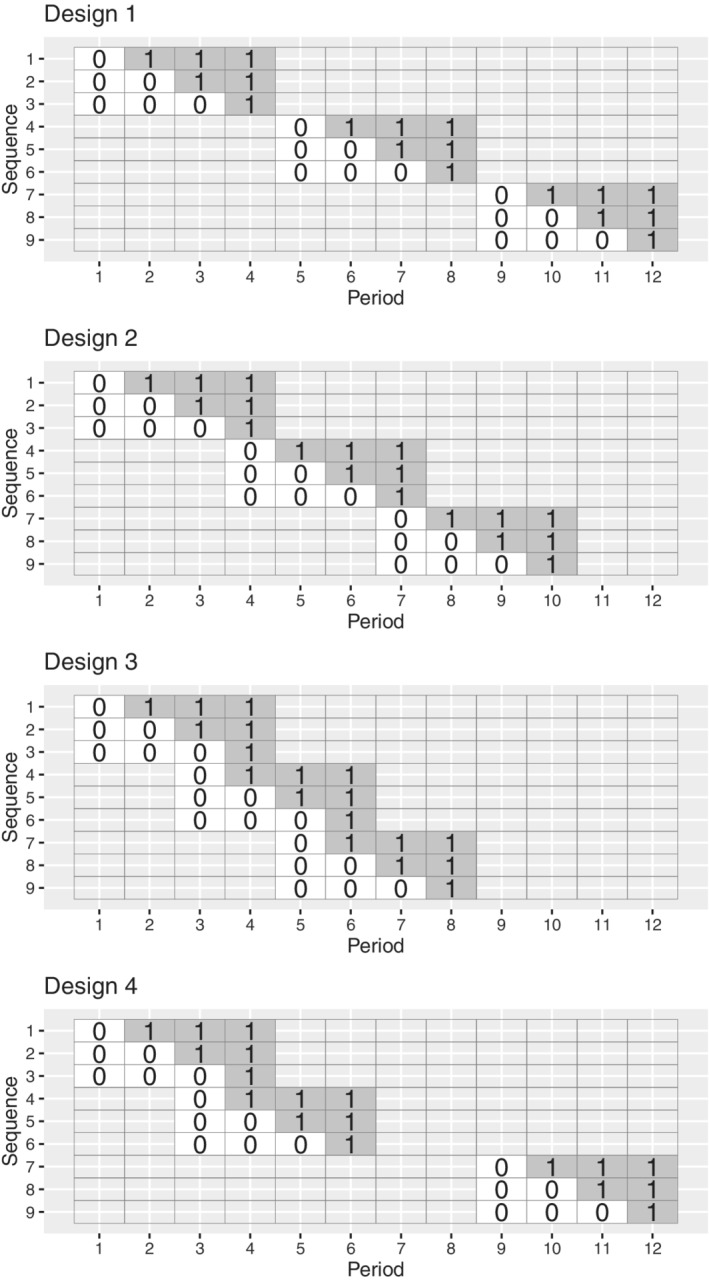
Four examples of batched stepped wedge designs with identical component designs (0 indicates control periods; 1 indicates intervention periods). Each of these designs has three batches of three‐period stepped wedge designs, with differing degrees of overlap between successive batches. Design 1 (top row): no overlap between successive batches; Design 2 (second from top): overlap of one period between successive batches; Design 3 (second from bottom): overlap of two periods between successive batches; Design 4 (bottom row): variable overlap between successive batches

**FIGURE 4:**
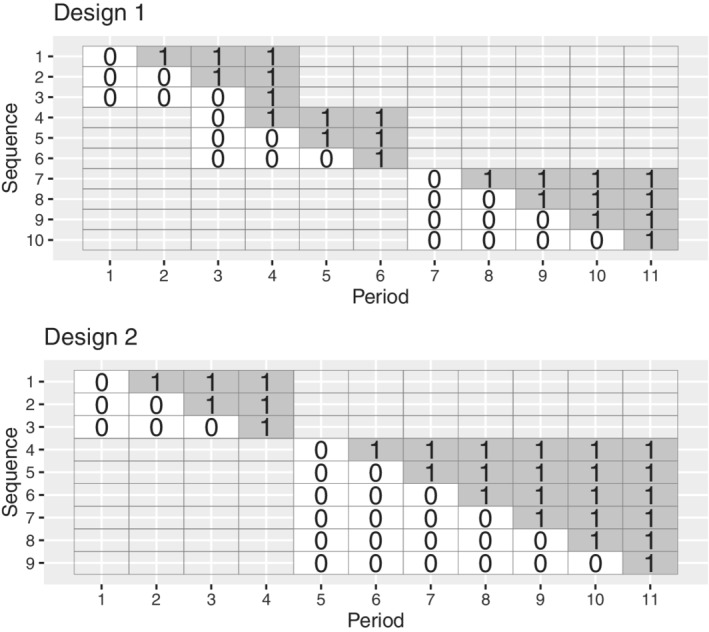
Two examples of batched stepped wedge designs without identical component designs (0 indicates control periods; 1 indicates intervention periods)

In this article, we formalize the situation where different groups of clusters commence trial participation at different calendar times, defining the “batched stepped wedge design.” In the batched stepped wedge design, different groups of clusters commence participation in a stepped wedge trial at different times, in a “batched” structure. The models we consider allow for systematic differences between clusters that commence study participation at different time points, and for differences in the effects of calendar and time‐on‐trial across these batches. This batched stepped wedge design is an alternative to the standard stepped wedge design: the batched design shares some of the benefits of the stepped wedge but allows for randomization of clusters to stepped wedge trial sequences in batches or blocks. Like the standard stepped wedge, the batched stepped wedge design ensures that all clusters eventually receive the intervention; treatment switches are unidirectional (ie, the intervention is never removed once implemented); and the intervention is rolled out to each cluster in a randomized order. Examples when all batches are identical are shown in Figure 3; examples when batches differ are shown in Figure 4. This batched stepped wedge design can thus be conducted similarly to standard cluster randomized trials, where clusters may be randomized to the control or the intervention condition in groups as clusters are recruited to the trial, rather than all at once.

Although guidance for researchers and statisticians in sample size and power calculations for batched stepped wedge and related designs is lacking, researchers have already sought to implement designs similar to batched stepped wedge designs. For example, in a study assessing the impact of a mobility program for patients aged 60+ years across 8 veterans affairs hospitals in the USA on discharge destination of patients, Hastings et al^7^ sought to implement a batched stepped wedge design, with two batches of clusters. In reference to this batched structure, Hastings et al^7^ stated, “The full implications of a blocked randomization from a statistical perspective require further study.” Similarly, the EAGLE study,^8^ investigating the impact of a quality improvement intervention on the reduction of anastomotic leak following right colectomy, is randomizing hospitals in batches to a series of dog‐leg designs (the dog‐leg can be considered as an incomplete three‐sequence stepped wedge design).^9^

The batched stepped wedge design may be appealing to researchers for three reasons: (1) depending on assumptions made about the outcome regression model, the power of a batched stepped wedge design will be unaffected by delays to the commencement of subsequent batches; (2) it can allow trials to get started sooner, by allowing clusters to come on‐line in batches (ie, ethics approvals and data collection procedures can be rolled out across clusters after the study has commenced); and (3) it does not require data to be collected by all clusters in all periods (ie, this design can be thought of as an “incomplete” stepped wedge design. Expanding on the first reason: in this article, we show that for linear mixed models, if separate period effects are included for each batch of a batched stepped wedge (as in the bottom panel of Figure 2), then the power of the batched stepped wedge design is equivalent to the power of a standard stepped wedge design with multiple clusters assigned to each sequence. We also show that this will also hold when further assumptions about the effect of time are made when binary outcomes are analyzed using nonlinear link functions and generalized estimating equations. That is, under these assumptions, the designs in Figure 3 would have equivalent power to detect a difference as the design in Figure 1 with 3 clusters per sequence—although we would encourage trialists to include more than 9 clusters in any stepped wedge trial.^10^

In this article, we provide researchers with guidance regarding the statistical aspects of batched stepped wedge designs, making recommendations regarding the inclusion of batch and period effects in the outcome regression model. In Section 2, we describe the batched stepped wedge design; in Section 3, we consider sample size calculations for the batched stepped wedge assuming linear mixed models for outcomes; in Section 4, we consider binary outcomes modeled with generalized linear models fit via generalized estimating equations. In Sections 3 and 4, we discuss what assumptions must be made in the specification of the outcome regression model to ensure the robustness property of the batched stepped wedge (where study power is robust to delays in the recruitment and/or commencement of the next batch) will hold. In Section 5, we discuss under what conditions standard stepped wedge sample size software can be applied to batched stepped wedge designs and demonstrate this calculation via an example. In Section 6, we present the results of a simulation study, and conclude with a discussion of our results in Section 7.

## 2 WHAT IS A BATCHED STEPPED WEDGE DESIGN?

Simply put, a batched stepped wedge design is a series of stepped wedge cluster randomized trials. There may be some overlap in time between the successive stepped wedge components of the batched stepped wedge design, that is, some trial periods during which data is being collected from more than one batch of the design. The component stepped wedge trials may be identical (as in the batched stepped wedge designs in Figure 3), or they may differ (as in Figure 4). Different sets of clusters contribute data in different batches of the study, and within each batch, clusters are randomized to the different sequences of the component stepped wedge design. A batched stepped wedge design allows for the recruitment of clusters throughout the duration of a study: once enough clusters for one of the component stepped wedges have been recruited, these clusters can be randomized to the sequences of the next batch, and the next stepped wedge component can commence. The models that we propose account for systematic differences between the clusters in different batches.

In a standard stepped wedge design, the implicit assumption is that all clusters commence participation in the trial at the same time^11^ (or that there are assumptions made regarding the effect of time‐on‐trial as discussed in Section 1). This is in contrast to the way in which parallel, or standard, cluster randomized trials are conducted. When parallel cluster randomized trials are conducted, clusters are often recruited throughout the duration of the trial. As is well known, in the (unstratified) parallel cluster randomized trial, so long as equal numbers of clusters (with equal numbers of participants) are assigned to the control and intervention arms at each randomization point, this successive recruitment has no impact on the power of the study. This also holds for cluster randomized crossover designs provided equal numbers of clusters with equal numbers of participants implement the control and the intervention arm at each time point (again, this observation is limited to unstratified designs). The reason for this is that for these parallel and cluster randomized trial designs, treatment condition and time are independent: at each time point of the study, half of the clusters and participants will be in the control condition, and the other half will be in the intervention. In the stepped wedge design, treatment and time are not independent: the proportion of clusters in the intervention condition increases as time passes.^2^ Depending on how time and randomization batch are accounted for in the outcome regression model used to inform sample size calculations, the batched randomization could have an impact on study power for batched stepped wedge designs, due to the confounding of time and treatment.

It is now well‐recognized that time/period effects need to be accounted for in sample size and power calculations for stepped wedge designs.^3^ Time/period effects must similarly be accounted for in sample size calculations for batched stepped wedge designs; researchers must provide adequate justification if they do not account for period effects in this calculation. Further, clusters that are included in different batches of the design may differ from each other, and thus it is recommended that batch effects be included in the outcome regression model. When there is an overlap between batches (eg, the middle and bottom panels of Figure 3; the top panel of Figure 4), we also recommend that separate fixed period effects be included for each batch (equivalent to fixed batch‐by‐period interaction terms being included in the model). There are three key reasons for this recommendation: the first is that it requires making the fewest assumptions about the effects of time on outcomes and whether these effects are shared across batches; the second is that under this assumption, the variance of the treatment effect estimator for the batched stepped wedge is a combination of the variance for each component stepped wedge; the third (and most important) is that under this assumption, in many situations, study power will be robust to delays in the commencement of batches

We now consider the variance of the treatment effect estimator for batched stepped wedge designs. We first consider linear mixed models in Section 3, discussing batched stepped wedge designs with identical and nonidentical components separately, and then discuss generalized linear models fit via GEE in Section 4.

## 3 BATCHED STEPPED WEDGE DESIGNS AND LINEAR MIXED MODELS

### 3.1 Batched stepped wedges with identical components

We first suppose that the batched stepped wedge design being considered is composed of B batches of identical T‐period and K‐sequence designs (for the standard stepped wedge K=T−1), and initially suppose that one cluster is assigned to each sequence of each batch. We consider the following linear mixed model for the outcome $Y_{bkti}$ from participant i=1,…,m in period t=1,…,T from cluster k=1,…,K in batch b=1,…,B:

(1) $$\begin{array}{ll} Y_{bkti}=\beta_{bt}+\theta X_{bkt}+\alpha_{bkt}+\epsilon_{bkti},\;\epsilon_{bkti}∼N(0,\sigma_{\epsilon }^{2}). & \end{array}$$

In this model, we have numbered the periods within each batch separately and thus period is identical to time‐on‐trial: $Y_{11Ti}$ represents the outcome for the ith participant in cluster 1 in the final period (period T) of the first batch. If there is one period of overlap between successive batches, period T of batch 1 would correspond to period 1 of batch 2. Our model set‐up automatically allows for separate period effects in each batch through the inclusion of the $\beta_{bt}$ period terms (ie, the scenario in the bottom panel of Figure 2): there are B×T period terms in total. These fixed period effects could alternatively be parameterized as period effects (where the effect for each period is shared by all clusters contributing data in that period, no matter their batch), batch effects, and terms for the period‐by‐batch interaction. This would require either constraining some of the $\beta_{bt}$ to be identical, or renumbering period from 1 to the total number of periods in the entire study (eg, in Design 1 of Figure 3, the period subscript would range from 1 to 12; in Design 2 of Figure 3, the period subscript would range from 1 to 10). Given that later in this article, we recommend that separate period effects be included for each batch, throughout this article we will number period within each batch (conceiving of time as time‐on‐trial; although this distinction from calendar time is immaterial when including an interaction with the batch term).

The treatment effect of interest is θ, assumed to be constant across batches, and the treatment group of cluster k in batch b at time period t is indicated by the binary variable $X_{bkt}$. The T‐length vector of random effects $\alpha_{bk}={\left(\alpha_{bk1},\ldots ,\alpha_{bkT}\right)}^{T}$ for cluster k in batch b is assumed to have a multivariate normal distribution, centered around zero, with a variance matrix such that $\mathrm{var}(\alpha_{bkt})=\sigma_{\alpha }^{2}$ and $\text{cov}(\alpha_{bkt},\alpha_{bks})=r_{ts}\sigma_{\alpha }^{2}$, with $0\leq r_{ts}\leq 1$. If $r_{ts}=r^{\left|t-s\right|}$ for some 0<r<1, the discrete‐time decay model of^12^ is returned; if $r_{ts}=r$ for some 0<r≤1, the nested exchangeable model is returned, with r=1 corresponding to the Hussey and Hughes model.^4^

It is mathematically convenient to collapse Model 1 to cluster‐period means when investigating the statistical power of designs:^13^

(2) $$\begin{array}{ll} Y_{bkt}=\frac{1}{m}\overset{m}{\underset{i=1}{\sum }}Y_{bkti}=\beta_{bt}+\theta X_{bkt}+\alpha_{bkt}+\epsilon_{bkt},\;\epsilon_{bkt}∼N\left(0,\frac{\sigma_{\epsilon }^{2}}{m}\right). & \end{array}$$

In the following result, we consider the variance of the treatment effect estimator for models of the form given in Equations (1) and (2).

Result 1 Suppose that each batch of the batched stepped wedge design is identical, and models of the form in Equations (1) and (2) are considered, so that the cluster‐period means from each cluster share a common variance matrix, denoted by V. V is a T×T matrix, with the (t,s) element given by $\text{cov}(Y_{bkt},Y_{bks})$. If $X_{bk}$ is the T×1 vector containing the treatment indicators of cluster k in batch b for all T periods, then $X_{bk}=X_{b^{\prime }k}=X_{k}$ for all pairs of batches b and $b^{\prime }$, and the variance of the treatment effect estimator $\hat{\theta }$ is given by:

(3) $$\begin{array}{ll} \mathrm{var}(\hat{\theta })=\frac{1}{B}{\left[\overset{K}{\underset{k=1}{\sum }}X_{k}^{T}V^{-1}X_{k}-\frac{1}{K}\left(\overset{K}{\underset{k=1}{\sum }}X_{k}^{T}V^{-1}\overset{K}{\underset{k=1}{\sum }}X_{k}\right)\right]}^{-1}=\frac{1}{B}{\mathrm{var}}_{0}(\hat{\theta }), & \end{array}$$

where ${\mathrm{var}}_{0}(\hat{\theta })$ is the variance of the treatment effect estimator for one of the components of the batched design with one cluster per sequence. This result can be generalized to the situation where $C_{b}$ clusters are assigned to each sequence of batch b. When this is the case,

(4) $$\begin{array}{ll} \mathrm{var}(\hat{\theta })=\frac{1}{\sum_{b=1}^{B}C_{b}}{\mathrm{var}}_{0}(\hat{\theta }). & \end{array}$$

Result 1 indicates that when batch‐by‐time interaction terms are included in the model for the outcome, the treatment effect estimator is simply a weighted combination of treatment effect estimators obtained from each batch separately. Specifically, the estimator from each batch is weighted by its variance.

Result 1 is a consequence of the more general result discussed in Section 3.2, with the proof provided in Section 1 of the Supplementary Material available online. Equation (3) indicates that the variance of the treatment effect estimator from the batched stepped wedge design with B batches of identical T‐period stepped wedge designs on K clusters is equivalent to the variance of the treatment effect estimator for a single T‐period stepped wedge design with B×K clusters. When $C_{b}$ clusters are assigned to each sequence of batch b, the variance of the treatment effect estimator for the batched design is equivalent to that of the single component design with $\sum_{b=1}^{B}C_{b}$ clusters per sequence. When all batches are identical and a model such as that in Equation (1) is assumed, sample size calculations for batched stepped wedge designs are thus straightforward. We demonstrate such calculations in Section 5.

Model 1 can be extended to allow for closed or open cohort schemes (eg, as described in Kasza et al^14^), to incorporate treatment effect heterogeneity (eg, as described in Kasza et al^15^), and to allow for differing numbers of subjects in each cluster in each period (eg, as described in Kasza et al^16^). When treatment effect heterogeneity is included in the model, the variance matrix V will not be identical across the clusters within a batch. However, the variance of the treatment effect estimator for a batch (denoted by ${\mathrm{var}}_{0}(\hat{\theta })$ in Equation (3) will be common across batches. Hence when treatment effect heterogeneity is included in the model, the variance of the treatment effect estimator from the batched stepped wedge design with B batches of identical component designs with $C_{b}$ clusters per sequence in batch b is again equivalent to the variance of the treatment effect estimator for a single component with $\sum_{b=1}^{B}C_{b}$ clusters per sequence.

When different clusters have different numbers of participants in each cluster‐period, there may not be a common joint variance matrix ${\mathrm{var}}_{0}(\hat{\theta })$ across batches. When cluster sizes differ, but each cluster is expected to collect the same number of observations in each of their data collection periods (ie, cluster k collects $m_{k}$ observations in each period), researchers could calculate the mean and coefficient of variation of cluster sizes and use the approximation presented in Reference 17 to obtain a common ${\mathrm{var}}_{0}(\hat{\theta })$ for each batch of the design.

### 3.2 Batched stepped wedges with nonidentical components

We now consider batched stepped wedge designs with nonidentical components (examples in Figure 4): we suppose that there are B batches of stepped wedge designs, where batch b is a $T_{b}$‐period stepped wedge design, with $K_{b}$ clusters. Result 2 provides the variance of the treatment effect estimator when batches are no longer identical.

Result 2 If $Y_{bkti}$ is the outcome for participant $i=1,\ldots ,m_{bkt}$ in period $t=1,\ldots ,T_{b}$ in cluster $k=1,\ldots ,K_{b}$ in batch b=1,…,B, and $Y_{b}$ is the $M_{b}=\sum_{k=1}^{K_{b}}\sum_{t=1}^{T_{b}}m_{bkt}$‐length vector of outcomes from all clusters in batch b, we suppose that

$$Y_{b}∼N(Z_{b}\gamma_{b}+\theta X_{b},\sum_{b}),$$

where $\gamma_{b}$ is the $T_{b}$‐length vector of period effects for batch b, $Z_{b}$ is the design matrix associated with these period effects for cluster b (of dimension $M_{b}\times T_{b}$), θ is the treatment effect of interest (assumed to be shared across all batches), $X_{b}$ is the $M_{b}$‐length vector indicating if a participant is in a cluster‐period in the control condition ($X_{bkti}=0$) or the intervention condition ($X_{bkti}=1$), and $\sum_{b}$ is the $M_{b}\times M_{b}$ covariance matrix of the outcomes from all clusters in batch b. Then if $\hat{\theta }$ is the generalized least squares estimator of θ,

(5) $$\begin{array}{ll} \mathrm{var}(\hat{\theta })={\left(\overset{B}{\underset{b=1}{\sum }}\frac{1}{{\mathrm{var}}_{b}(\hat{\theta })}\right)}^{-1}, & \end{array}$$

where ${\mathrm{var}}_{b}(\hat{\theta })$ is the variance of the generalized least squares estimator of θ obtained by considering batch b only. Further, if ${\mathrm{var}}_{b}(\hat{\theta })={\mathrm{var}}_{0}(\hat{\theta })$ then $\mathrm{var}(\hat{\theta })=\frac{1}{B}{\mathrm{var}}_{0}(\hat{\theta })$.

The proof of Result 2 is provided in Section 1 of the Supplementary Material. This result assumes normally distributed outcomes where only the treatment effect is shared across batches; no assumptions are made regarding the equality of within‐cluster correlation structures within or between batches. However, if ${\mathrm{var}}_{b}(\hat{\theta })={\mathrm{var}}_{0}(\hat{\theta })$ for some ${\mathrm{var}}_{0}(\hat{\theta })$ for all b=1,…,B then Equation (5) collapses to the result given in Result 1, that is, the situation where all batches are identical. Once again, the treatment effect estimator is the weighted sum of the estimators obtained for each batch, with each batch's estimator weighted by its variance.

## 4 BINARY AND COUNT OUTCOMES AND BATCHED STEPPED WEDGES

Several modeling options are available when researchers are interested in binary, rather than continuous outcomes. One option, discussed in Hussey and Hughes,^4^ and taken in Hemming et al,^5^ is to apply Equation (1) to binary outcomes, setting $\sigma_{\epsilon }^{2}$ equal to p(1−p), where $p=P(Y_{bkti}=1)$. The generalized least squares estimator of θ is then considered, and results in Section 3 apply. However, it has been pointed out that the variance of the treatment effect estimator may not be adequately approximated when this approach is applied.^18^ Zhou et al^18^ developed an alternative approach, assuming a truncated normal distribution for cluster random effects, to ensure that estimated probabilities lie between 0 and 1. When batch and period are parameterized as in Equation (1) (ie, separate period effects are included for each batch in the outcome regression model), the variance of the treatment effect estimator can be written as the sum of variances for each stepped wedge component as for the continuous outcome.

When binary or count outcomes are of interest, researchers are frequently interested in estimating a marginal treatment effect instead of the conditional treatment effect. The use of generalized estimating equations (GEE) for the analysis of longitudinal cluster randomized trials allows for estimation of such marginal effects, and implications of this analysis approach for sample size calculations have previously been discussed.^6^, ^19^ When the GEE approach is used, a working correlation matrix structure must be assumed. This working correlation structure describes the pattern of within‐cluster correlations; an exchangeable correlation structure would imply equal correlations between all observations in a cluster, for example. As discussed in Li et al,^6^ when GEE is the intended analysis approach, power calculations can proceed via generalized least squares. We now state the main result for this scenario.

Result 3 If $Y_{bkti}$ is the outcome for participant $i=1,\ldots ,m_{bkt}$ in period $t=1,\ldots ,T_{b}$ in cluster $k=1,\ldots ,K_{b}$ in batch b=1,…,B, with $\mu_{bkti}=E[Y_{bkti}]$, we assume

(6) $$\begin{array}{ll} g(\mu_{bkti})=\beta_{bt}+\theta X_{bkt}, & \end{array}$$

where g is the link function, $\beta_{bt}$ is the fixed effect for period t in batch b, θ is the treatment effect of interest, and $X_{bkt}$ is the indicator for whether cluster k in batch b and period t is in the intervention or control condition. Let $\mu_{b}$ be the vector of means for batch b. If $\beta_{b}={\left(\beta_{b1},\ldots ,\beta_{bT_{b}}\right)}^{T}$ is the set of time effects for batch b, ${\hat{\beta }}_{b}$ is the generalized least squares estimator of $\beta_{b}$, and $\hat{\theta }$ is the generalized least squares estimator of θ then

(7) $$\begin{array}{ll} \mathrm{var}(\hat{\theta }) & ={\left(\overset{B}{\underset{b=1}{\sum }}{\frac{\partial \mu_{b}}{\partial \hat{\theta }}}^{T}W_{b}^{-1}\frac{\partial \mu_{b}}{\partial \hat{\theta }}-{\frac{\partial \mu_{b}}{\partial \hat{\theta }}}^{T}W_{b}^{-1}\frac{\partial \mu_{b}}{\partial \hat{\beta_{b}}}{\left[{\frac{\partial \mu_{b}}{\partial \hat{\beta_{b}}}}^{T}W_{b}^{-1}\frac{\partial \mu_{b}}{\partial \hat{\beta_{b}}}\right]}^{-1}{\frac{\partial \mu_{b}}{\partial \hat{\beta_{b}}}}^{T}W_{b}^{-1}\frac{\partial \mu_{b}}{\partial \hat{\theta }}\right)}^{-1}\\ & ={\left(\overset{B}{\underset{b=1}{\sum }}\frac{1}{{\mathrm{var}}_{b}(\hat{\theta })}\right)}^{-1}, \end{array}$$

where ${\mathrm{var}}_{b}(\hat{\theta })$ is the variance of the treatment effect estimator obtained via GEE when batch b is considered separately, and $W_{b}$ is the covariance matrix of the observations from batch b. $W_{b}$ has the form $A_{b}^{1/2}R_{b}A_{b}^{1/2}$. $A_{b}$ is a diagonal matrix with elements given by $\mathrm{var}(Y_{bkti})$ and $R_{b}$ is the assumed correlation matrix of the observations from batch b. If a binomial distribution for outcomes is assumed $\mu_{bkti}=P(Y_{bkti}=1)$ and diagonal elements of $A_{b}$ will be given by $\mathrm{var}(Y_{bkti})=\mu_{bkti}(1-\mu_{bkti})$.

The proof of this result is shown in the Appendix. As was the case in the linear mixed model scenario, the treatment effect estimator is again a weighted sum of each batch's treatment effect estimator. However, in contrast to the linear model discussed in Section 3, the variance of the treatment effect estimator depends on the assumed period effects through $\mu_{bkti}$ in the model in Equation (6). For example, when calculating sample sizes for a batched stepped wedge trial where a marginal model will be used to analyze binary outcomes, researchers must include predicted prevalences of the outcome in each period of the trial in sample size calculations. For binary outcomes that will be analyzed in this way, the variance of the batched stepped wedge design will collapse to the simplified form given in Equation (4) only if no period effects are included in the model. This is a strict assumption, requiring the prevalence of the outcome in the control arm to be the same for the entire trial duration and across batches (ie, there are no secular time effects and no batch effects).

Result 3 applies not only to binary outcomes analyzed with a logit link function; the proof given in the Appendix does not rely on the choice of link function or the outcome type. Thus, Result 3 holds for binary outcomes with a linear link function, or for count outcomes with a log link function, to name just two alternatives.

## 5 DEMONSTRATION OF POWER CALCULATIONS FOR BATCHED STEPPED WEDGE DESIGNS

**FIGURE 5:**
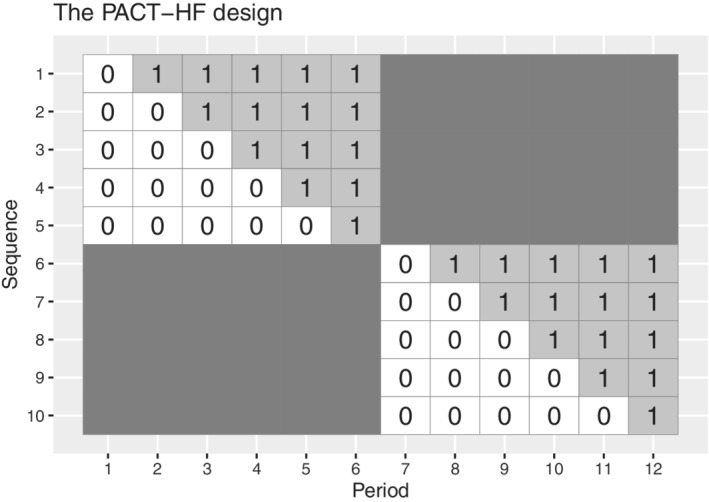
The design schematic for the PACT‐HF trial: Two batches of a 5‐sequence stepped wedge design

We demonstrate how to calculate power for a batched stepped wedge design with two identical batches. The example we consider is from Unni et al:^20^ in that paper, various different stepped wedge‐like designs were considered for the Patient‐Centered Care Transitions in Heart Failure Trial (PACT‐HF), including a batched design with “early” and “late” blocks, shown in Figure 5. Each of these blocks was a 5‐sequence, 6‐period stepped wedge design, with one cluster assigned to each sequence, with 54 patients in each cluster in each of these periods. The primary outcome considered was a binary outcome, that was a composite of a number of clinical outcomes, with a prevalence of 28% under the control condition, and an intracluster correlation of 0.01. We assume that this intracluster correlation was conditional on the inclusion of a “batch” term in the model (a point we return to in Section 7), but in practice we would recommend assessing the impact of varying this correlation on study power. The aim was to detect a 25% reduction in the prevalence of the outcome: that is, a reduction from 28% to 21%. The effect on the logit link scale is −0.38.

We consider two methods for calculating the power of this design: first, we assume a linear model for the binary outcome (applying the results of Section 3.1); second, we assume that a GEE approach will be taken to fitting a model with a logit link (applying the results of Section 4).

To perform the power calculation for the first approach, one need only calculate the power of a standard 6‐period stepped wedge design with 2 clusters assigned to each sequence. The Shiny CRT calculator^5^ accommodates this by allowing users to set the number of clusters assigned to each sequence; the Stata steppedwedge program^21^ accommodates this through the “k” option. When the linear model is assumed, this study has a power of 77% to detect the difference. There are two additional ways to use the Shiny CRT calculator to calculate the power of the batched design. The user could get the precision of each of the component designs separately using the “Precision” tab on the Shiny CRT calculator, and then combine these according to Result 1. Alternatively, the user could upload the design matrix for the batched design (ensuring that there is no overlap between successive batches) and obtain the power of the design directly. Were a design matrix uploaded with an overlap between successive batches, the Shiny CRT calculator would assume that batches with overlapping periods share period effects (that is, calendar time effects would be shared across batches as in the top panel of Figure 2).

For the second method we use the R swdpwr package^22^ to calculate the power of the batched stepped wedge design. Since the GEE approach depends on the baseline prevalence of the outcome, we consider two different scenarios:

1. The prevalence of the outcome under the control condition remains at 28% for the entire duration of the trial.
2. The prevalence of the outcome under the control condition is initially 30%, but decreases to 28% by the final period of the trial, in a linear fashion. That is, at the time that the second batch starts data collection, the prevalence of the outcome under the control condition is 29%.

Since there is no change in the underlying prevalence of the condition over time in the first scenario, the power of the batched stepped wedge using the GEE approach is equivalent to the power of the 6‐period stepped wedge with 2 clusters assigned to each sequence. The power of this design can be obtained directly by using the swdpower command in the swdpwr package, and is 98.8%. Power is high due to the omission of period effects in this calculation.

For the second scenario, the variance of the treatment effect estimator must be obtained for each of the two component designs separately. We assume that the treatment effect is −0.38 on the logit link scale for both batches, with the aim to detect a reduction from 29% to 21.75% for the first batch, and from 28% to 21% in the second batch. The swdpower command cannot provide the power of the batched design directly. However, the variance of the treatment effect estimator can be obtained for each batch separately from the power calculated by the command. These variances are then combined using Result 3. The power of the batched design to detect a change from 28% to 21% is 80.8%. Commands to replicate this calculation are provided in Section 2 of the Supplementary Material.

## 6 SIMULATION STUDY

We conducted a simulation study to verify our theoretical results, inspired by the PACT‐HF design discussed in Section 5. As in the design schematic in Figure 5, we consider a design consisting of two batches, each a 6‐period stepped wedge design. However, we vary the number of overlapping periods between the two batches from 0 (indicating no overlap between the two batches, as in Figure 5) to 5 (batches that overlap completely); the key aim of this simulation study is to assess whether inclusion of separate period effects for each batch has an impact on empirical power. Does power decrease as the number of batch‐by‐time terms in the model increases? In the simulation we increase the total number of clusters to 40 (4 clusters assigned to each of the 10 sequences). We simulate both binary and continuous outcomes for a range of correlation parameter values. Code to replicate this simulation study and the nested loop plots is available at https://github.com/jkasza/BatchSW.

Table 1 lists the parameters considered for the simulation study for the continuous outcomes. Along with varying the number of periods of overlap between successive batches, the intracluster correlation and the cluster autocorrelation, datasets were simulated with an effect size of 0 (to allow an examination of significance level) and 0.15. For each combination of parameters in Table 1, 1000 datasets were simulated, with separate time effects in each batch (as in the bottom panel of Figure 2). The period effects for each batch in each period were simulated from a normal distribution with mean 0 and variance 1. With 1000 simulated datasets, the Monte Carlo standard error associated with a power of 80% is expected to be around ±1.3%.^23^ Each simulated dataset was analyzed using a linear mixed‐effects model with random effects for cluster and cluster‐period, and separate categorical fixed period effects for each batch (ie, period effects, batch effects, and period by batch interaction terms). Our focus here is on the comparison of theoretical and simulated power, so for each set of parameters, we calculated the percentage of hypothesis tests $H_{0}:\theta =0$ rejected at the two‐sided 5% significance level.

**TABLE 1:** The continuous outcome simulation settings

Parameter	Meaning	Values
T	Number of periods in each stepped wedge design	6
B	Number of batches	2
K	Number of clusters assigned to each sequence	4
m	Number of observations in each cluster in each sequence	10
$N_{O}$	Number of periods of overlap between successive batches	5, 4, 3, 2, 1, 0
ρ	Intra‐cluster correlation	0.01, 0.05, 0.1
r	Cluster autocorrelation	1, 0.95, 0.75
θ	Effect size	0, 0.15

*Note*: One thousand datasets were simulated for each combination of parameters (108 combinations).

**FIGURE 6:**
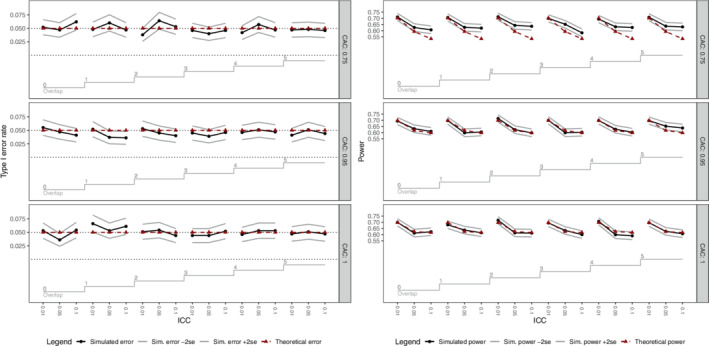
Empirical and theoretical type I error rates (left panel) and power (right panel) for the simulated continuous outcomes. ICC, intracluster correlation; CAC, cluster autocorrelation. Within each panel, subpanels correspond to a different value of the CAC. The theoretical and empirical type I error rate or power is displayed for each combination of number of periods of overlap, ICC, and CAC, with the empirical result plus and minus 2 standard errors also displayed

Figure 6 displays the empirical type I error rates and power for each set of parameters using nested loop plots.^24^ This figure indicates that the number of periods of overlap does not have an impact on empirical type I error rates and power: as the number of periods of overlap changes, there is no pattern to the variation in empirical type I error rates or power. This provides support for our theoretical result, which indicates that if period, batch, and batch by period interaction terms are included in the model, the degree of overlap has no impact on study power. As expected, Figure 6 does indicate that the intracluster correlation and cluster autocorrelation do have an impact on empirical power levels. It is interesting to note that for some combinations of the cluster autocorrelation and intracluster correlation (eg, when the cluster autocorrelation is 0.75, and the intracluster correlation is equal to 0.05 or 0.1), the empirical power is slightly inflated. However, this does not change as the overlap between batches decreases. That is, empirical power does not decrease as the number of time effects included in the model increases.

**FIGURE 7:**
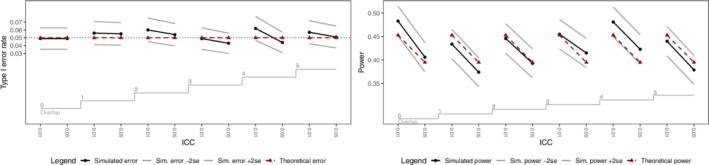
Empirical and theoretical type I error rates (left panel) and power (right panel) for the simulated binary outcomes analyzed via GEE. ICC, intracluster correlation. The theoretical and empirical type I error rate or power is displayed for each combination of number of periods of overlap and ICC, with the empirical result plus and minus 2 standard errors also displayed

Table 2 lists the parameters considered for the simulation study for the binary outcomes. As was the case for the simulation study for continuous outcomes, 1000 datasets were simulated for each combination of parameters. Binary data was simulated using the method of Qaqish,^25^ as coded by Li et al.^19^ The range of intracluster correlations permitted by the simulation method of Qaqish is limited, so we only consider intracluster correlations of 0.01 and 0.05 for the binary outcomes. Each simulated dataset was analyzed via GEE with a logit link with an exchangeable working correlation structure, with separate coefficients for period (treated as a continuous covariate) in each batch (this choice is to match the sample size calculation in the R swdpwr package^22^). Again, our focus is on the comparison of theoretical and simulated power, so for each set of parameters and each analysis choice, we calculated the percentage of hypothesis tests $H_{0}:\theta =0$ rejected at the two‐sided 5% significance level. Theoretical power for each combination of parameters was also calculated, using the swdpwr package. Figure 7 displays the empirical type I error rates and power for each set of parameters for the binary outcomes analyzed via GEE with an exchangeable working correlation. As for the simulation study for continuous outcomes, the simulated power and type I error rates do not depend on the degree of overlap between successive batches, aligning with our theoretical results.

**TABLE 2:** The binary outcome simulation settings

Parameter	Meaning	Values
T	Number of periods in each stepped wedge design	6
B	Number of batches	2
K	Number of clusters assigned to each sequence	4
m	Number of observations in each cluster in each sequence	10
$P(Y_{bkt}=1\;|\;X_{bkt}=0)$	Probability of the outcome in a nontreatment period	0.4+t×0.01
$N_{O}$	Number of periods of overlap between successive batches	5, 4, 3, 2, 1, 0
ρ	Intra‐cluster correlation	0.01, 0.05
r	Cluster autocorrelation	1
$P(Y_{bkt}=1\;|\;X_{bkt}=1)-P(Y_{kt}=1\;|\;X_{kt}=0)$	Change in probability of the outcome caused by the intervention	0,0.025

*Note*: One thousand datasets were simulated for each combination of parameters (24 combinations).

## 7 DISCUSSION

The batched stepped wedge design is a promising alternative to the standard stepped wedge design. By allowing clusters to come on‐line to the study in batches, the batched stepped wedge design has the potential to get started sooner than a standard stepped wedge, which typically requires all clusters to commence at the same point in time. If separate period effects are included for each batch (as in the bottom panel of Figure 2), the power of the batched stepped wedge design will, depending on the assumed outcome regression model, be robust to delays in the commencement of batches. This holds when linear models for the outcome are assumed, or when the prevalence of the outcome in the control condition is not expected to change over time. Hence, in these settings, study power will be unaffected if there is an unanticipated delay before the next batch commences study involvement when separate period effects are assumed for each batch. Under this assumed model, standard stepped wedge software can be used to calculate the required sample size and study power for batched stepped wedge designs.

Our key result indicates that in certain situations a batched stepped wedge design consisting of B identical stepped wedge designs provides the same power to detect an effect as one of the stepped wedge components with B clusters assigned to each sequence. However, the choice of variance components will have an impact on sample size and power calculations for all batched stepped wedge designs. Inclusion of the batch term implies that variance components must now be treated as “within‐batch” variance components, and will likely be smaller than if a model without batch effects was considered. When batch effects are included in the outcome regression model, variance components will be conditional on the inclusion of these batch effects in the model. Hence, researchers must consider the impact of batches on variance components and intracluster correlations when considering sample size and power.

Our key results have broad applicability. They generalize to batches of any other type of longitudinal cluster randomized trial design and do not rely on the design type. For example, our results apply to a “batched dog‐leg” design. Provided that separate period effects are included for each batch, the variance of such a design would have the form given in Sections 3 and 4: summing over the variances of treatment effect obtained for each of the individual component designs. Further, our key results do not depend on the precise form of the variance of the treatment effect estimator for each batch. The models considered in the Results could be extended to allow for closed or open cohorts, treatment effect heterogeneity, etc. The key result only requires that separate period effects are included in the outcome model for each batch: if this is the case, then the variances of the treatment effect estimators for each batch can be combined according to Results 1, 2, or 3 as appropriate.

We recommend that separate period effects are included for each stepped wedge batch (ie, the time parameterization as in the bottom panel of Figure 2) to provide robustness to the sample size calculation in case of unexpected recruitment and set‐up delays. In addition to allowing for robustness to delays and making the fewest assumptions about the effect of time on outcomes, assuming separate period effects across batches would be appropriate for trials where clusters in different batches are from geographically distinct areas, or where clusters in different batches are otherwise distinct. Additionally, if batch effects are not included in the outcome regression model and batches are assumed to have shared period effects for overlapping periods, then study power will depend on the separation between successive batches. If unexpected delays between batches occur, the power of the study will not be robust to this change, in that it will differ from that calculated a priori. Future work will investigate the impact of increasing degrees of overlap between successive batches when period effects are shared across batches.

Adaptive variants of the batched stepped wedge design are a logical next step of this work. Such adaptations may include sample size re‐estimation, or more formal stopping rules for efficacy or futility of the intervention based on assessments at suitable time points, for example, after participants in each batch have completed their followup. While adaptive variants of the stepped wedge design have been discussed in the literature, these do require that all clusters commence data collection at the same time. These adaptive variants will be explored in future work.

## CONFLICT OF INTEREST

The authors declare no potential conflict of interests.

## Supporting information


**Appendix S1** Supplementary material

## Data Availability

Results of simulations and the code to replicate the simulation study in Section 6 is available at https://github.com/jkasza/BatchSW.
