# Supplementary material for: The batched stepped wedge design: A design robust to delays in cluster recruitment
Source: Stat Med. 2022 May 21;41(18):3627–41. doi: 10.1002/sim.9438 (PMC9541502; doi:10.1002/sim.9438)
Supplement: Supplementary file 1 — Appendix S1 Supplementary material [file SIM-41-3627-s001.pdf]

Appendix to "The batched stepped wedge design: a design robust to delays in cluster recruitment"

Jessica Kasza, Rhys Bowden, Richard Hooper, Andrew Forbes

jessica.kasza@monash.edu

School of Public Health and Preventive Medicine,

Monash University,

553 St Kilda Road, Melbourne 3004, Victoria, Australia

1 Proof of results

Result 1. *We consider the following linear mixed model for the outcome Y_{bkti} from participant $i = 1, \ldots, m$ in period $t = 1, \ldots, T$ from cluster $k = 1, \ldots, K$ in batch $b = 1, \ldots, B$:*

$$Y_{bkti} = \beta_{bt} + \theta X_{bkt} + \alpha_{bkt} + \epsilon_{bkti}, \quad \epsilon_{bkti} \sim N(0, \sigma_\epsilon^2). \tag{1}$$

The treatment effect of interest is θ, assumed to be constant across batches, and the treatment group of cluster k in batch b at time period t is indicated by the binary variable X_{bkt}. β_{bt} is the average outcome under the control condition in period t of batch b. The T-length vector of random effects $\alpha_{bk} = (\alpha_{bk1}, \ldots, \alpha_{bkT})^T$ for cluster k in batch b is assumed to have a multivariate normal distribution, centered around zero. We suppose that the vector of cluster-period means is a sufficient statistic for the treatment effect, and that the cluster-period means from each cluster share a common variance matrix, denoted by V. V is a $T \times T$ matrix, with the (t, s) element given by $cov(Y_{bkt}, Y_{bks})$.

Supposing that each batch of the batched stepped wedge design is identical, and X_{bk} is the $T \times 1$ vector containing the treatment indicators of cluster k in batch b for all T periods, then $X_{bk} = X_{b'k} = X_k$ for all pairs of batches b and b', the variance of the treatment effect estimator $\hat{\theta}$ is given by:

$$var(\hat{\theta}) = \frac{1}{B}\left[\sum_{k=1}^{K} X_k^T V^{-1} X_k - \frac{1}{K}\left(\sum_{k=1}^{K} X_k^T V^{-1} \sum_{k=1}^{K} X_k\right)\right]^{-1} = \frac{1}{B} var_0(\hat{\theta}), \tag{2}$$

where $var_0(\hat{\theta})$ is the variance of the treatment effect estimator for one of the components of the batched design

with one cluster per sequence. This result can be generalised to the situation where C_b *clusters are assigned* to each sequence *of batch b. When this is the case,*

$$var(\hat{\theta}) = \frac{1}{\sum_{b=1}^{B} C_b} var_0(\hat{\theta}). \tag{3}$$

Proof. The proof of this result follows directly from the proof of the more general Result 2, and is shown at the end of the proof of that result below. □

Result 2. *If* Y_{bkti} *is the outcome for participant* $i = 1, \ldots, m_{bkt}$ *in period* $t = 1, \ldots, T_b$ *in cluster* $k = 1, \ldots, K_b$ *in batch* $b = 1, \ldots, B$*, and* Y_b *is the* $M_b = \sum_{k=1}^{K_b} \sum_{t=1}^{T_b} m_{bkt}$*-length vector of outcomes from all clusters in batch b, we suppose that*

$$Y_b \sim N(Z_b \gamma_b + \theta X_b, \Sigma_b)$$

where γ_b *is the* T_b*-length vector of period effects for batch b,* Z_b *is the design matrix associated with these period effects for cluster b (of dimension* $M_b \times T_b$*),* θ *is the treatment effect of interest (assumed to be shared across all batches),* X_b *is the* M_b*-length vector indicating if a participant is in a cluster-period in the control condition (*$X_{bkti} = 0$*) or the intervention condition (*$X_{bkti} = 1$*), and* Σ_b *is the* $M_b \times M_b$ *covariance matrix of the outcomes from all clusters in batch b. Then if* $\hat{\theta}$ *is the generalised least squares estimator of* θ*,*

$$var(\hat{\theta}) = \left(\sum_{b=1}^{B} \frac{1}{var_b(\hat{\theta})} \right)^{-1}, \tag{4}$$

where $var_b(\hat{\theta})$ *is the variance of the generalised least squares estimator of* θ *obtained by considering batch b only. Further, if* $var_b(\hat{\theta}) = var_0(\hat{\theta})$ *then* $var(\hat{\theta}) = \frac{1}{B} var_0(\hat{\theta})$*.*

Proof. First write $Y = (Y_1, \ldots, Y_B)'$, the $\sum_{b=1}^{B} M_b$-length vector of all outcomes from the batched stepped wedge trial. Then we can write

$$Y \sim N(G\beta, \Sigma) \tag{5}$$

with

$$G = \begin{pmatrix} Z_1 & 0_{M_1 \times T_2} & \cdots & 0_{M_1 \times T_B} & X_1 \\ 0_{M_2 \times T_1} & Z_2 & \cdots & 0_{M_2 \times T_B} & X_2 \\ \vdots & \vdots & & & \\ 0_{M_B \times T_1} & 0_{M_B \times T_2} & \cdots & Z_B & X_B \end{pmatrix}, \quad \beta = \begin{pmatrix} \gamma_1 \\ \gamma_2 \\ \vdots \\ \gamma_B \\ \theta \end{pmatrix}, \quad \Sigma = \begin{pmatrix} \Sigma_1 & 0_{M_1 \times M_2} & \cdots & 0_{M_1 \times M_B} \\ 0_{M_2 \times M_1} & \Sigma_2 & \cdots & 0_{M_2 \times M_B} \\ \vdots & & \ddots & \\ 0_{M_B \times M_1} & 0_{M_B \times M_2} & \cdots & \Sigma_B \end{pmatrix} \tag{6}$$

where $0_{n\times m}$ is an $n \times m$ matrix of zeros. Then the generalised least squares estimator of β is given by:

$$\hat{\beta} = (G^T\Sigma^{-1}G)^{-1}G^T\Sigma^{-1}Y$$

and

$$var(\hat{\beta}) = (G^T\Sigma^{-1}G)^{-1}.$$

The blocked structure of G and Σ means that

$$G^T\Sigma^{-1}G = \begin{pmatrix} Z_1^T\Sigma_1^{-1}Z_1 & 0_{M_1\times M_2} & \cdots & 0_{M_1\times M_B} & Z_1^T\Sigma_1^{-1}X_1 \\ 0_{M_2\times M_1} & Z_2^T\Sigma_2^{-1}Z_2 & \cdots & 0_{M_2\times M_B} & Z_2^T\Sigma_1^{-1}X_2 \\ \vdots & & \ddots & & \vdots \\ 0_{M_B\times M_1} & 0_{M_B\times M_2} & \cdots & Z_B^T\Sigma_B^{-1}Z_B & Z_B^T\Sigma_1^{-1}X_B \\ X_1^T\Sigma_1^{-1}Z_1 & X_2^T\Sigma_2^{-1}Z_2 & \cdots & X_B^T\Sigma_B^{-1}Z_B & \sum_{b=1}^B X_b^T\Sigma_b X_b \end{pmatrix} \quad (7)$$

and $var(\hat{\theta})$ is the final entry in $(G^T\Sigma^{-1}G)^{-1}$

$$\begin{aligned} var(\hat{\theta}) &= \left\{\sum_{b=1}^B X_b^T\Sigma_b^{-1}X_b - \sum_{b=1}^B X_b^T\Sigma_b^{-1}Z_b\left(Z_b^T\Sigma_b^{-1}Z_b\right)^{-1}Z_b^T\Sigma_b^{-1}X_b\right\}^{-1} \\ &= \left\{\sum_{b=1}^B \left(X_b^T\Sigma_b^{-1}X_b - X_b^T\Sigma_b^{-1}Z_b\left(Z_b^T\Sigma_b^{-1}Z_b\right)^{-1}Z_b^T\Sigma_b^{-1}X_b\right)\right\}^{-1}. \end{aligned} \quad (8)$$

Note that $var_b(\hat{\theta}) = \left(X_b^T\Sigma_b^{-1}X_b - X_b^T\Sigma_b^{-1}Z_b\left(Z_b^T\Sigma_b^{-1}Z_b\right)^{-1}Z_b^T\Sigma_b^{-1}X_b\right)^{-1}$ (i.e. the variance of $\hat{\theta}$ were only batch b used to estimate θ).

Hence,

$$var(\hat{\theta}) = \left\{\sum_{b=1}^B var_b(\hat{\theta})^{-1}\right\}^{-1}. \quad (9)$$

If $var_b(\hat{\theta}) = var_0(\hat{\theta})$ for $b = 1, \ldots, B$, then

$$var(\hat{\theta}) = \left(Bvar_0(\hat{\theta})^{-1}\right)^{-1} = \frac{1}{B}var_0(\hat{\theta}). \quad (10)$$

□

Result 3. *If Y_{bkti} is the outcome for participant $i = 1, \ldots, m_{bkt}$ in period $t = 1, \ldots, T_b$ in cluster $k =$*

$1, \dots, K_b$ *in batch* $b = 1, \dots, B$*, with* $\mu_{bkti} = E[Y_{bkti}]$*, we assume*

$$g(\mu_{bkti}) = \beta_{bt} + \theta X_{bkt}. \tag{11}$$

where g *is the link function,* β_{bt} *is the fixed effect for period* t *in batch* b*,* θ *is the treatment effect of interest, and* X_{bkt} *is the indicator for whether cluster* k *in batch* b *and period* t *is in the intervention or control condition. Let* μ_b *be the vector of means for batch* b*. If* $\beta_b = (\beta_{b1}, \dots, \beta_{bT_b})^T$ *is the set of time effects for batch* b*,* $\hat{\beta}_b$ *is the generalised least squares estimator of* β_b*, and* $\hat{\theta}$ *is the generalised least squares estimator of* θ *then*

$$\begin{aligned} var(\hat{\theta}) &= \left(\sum_{b=1}^{B} \frac{\partial \mu_b}{\partial \hat{\theta}}^T W_b^{-1} \frac{\partial \mu_b}{\partial \hat{\theta}} - \frac{\partial \mu_b}{\partial \hat{\theta}}^T W_b^{-1} \frac{\partial \mu_b}{\partial \hat{\beta}_b} \left[\frac{\partial \mu_b}{\partial \hat{\beta}_b}^T W_b^{-1} \frac{\partial \mu_b}{\partial \hat{\beta}_b} \right]^{-1} \frac{\partial \mu_b}{\partial \hat{\beta}_b}^T W_b^{-1} \frac{\partial \mu_b}{\partial \hat{\theta}} \right)^{-1} \\ &= \left(\sum_{b=1}^{B} \frac{1}{var_b(\hat{\theta})} \right)^{-1} \end{aligned} \tag{12}$$

where $var_b(\hat{\theta})$ *is the variance of the treatment effect estimator obtained via GEE when batch* b *is considered separately, and* W_b *is the covariance matrix of the observations from batch* b*.* W_b *has the form* $A_b^{1/2} R_b A_b^{1/2}$*.* A_b *is a diagonal matrix with elements given by* $var(Y_{bkti})$ *and* R_b *is the assumed correlation matrix of the observations from batch* b*.*

If a binomial distribution for outcomes is assumed $\mu_{bkti} = P(Y_{bkti} = 1)$ *and diagonal elements of* A_b *will be given by* $var(Y_{bkti}) = \mu_{bkti}(1 - \mu_{bkti})$*.*

Proof. If Y_{bkti} is the outcome for participant i in period t in cluster k in batch b, then write $\mu_{bkti} = E[Y_{bkti}]$ and consider some link function g so that

$$g(\mu_{bkti}) = \beta_{bt} + \theta X_{bkt}.$$

Consider the vectors of all parameters (including the treatment effect θ) $\beta = (\beta_{11}, \beta_{12}, \dots, \beta_{1T}, \dots, \beta_{B1}, \beta_{B2}, \dots, \beta_{BT}, \theta)^T$, all observations Y and all means μ. Then, by [2] the GEE estimator for β is given by the solution to

$$D^T V^{-1}(Y - \mu) = 0 \tag{13}$$

where $D = \frac{\partial \mu}{\partial \beta^T}$, $V = A^{1/2} R A^{1/2}$ where R is the working correlation matrix and A has diagonal elements given by $\phi var(Y_{bkti})$. ϕ is a dispersion parameter; for our derivations we will assume that this is equal to 1. R is supposed to have a block-diagonal structure, with the blocks R_b corresponding to batches. That is, we only assume that observations in distinct batches are independent but make no assumptions about the supposed correlation within batches. The estimator $\hat{\beta}$ will be approximately normally distributed with mean

β and covariance matrix given by $(D^T V^{-1} D)^{-1}$. Of interest is the variance of $\hat{\theta}$, which corresponds to the element in the lower right hand corner of this matrix.

Since separate period effects are assumed for each batch in the model in Equation 11, we can write

$$g(\mu) = \begin{pmatrix} W_1 & & & & X_1 \\ & W_2 & & & X_2 \\ & & \ddots & & \vdots \\ & & & W_B & X_B \end{pmatrix} \begin{pmatrix} \beta_1 \\ \beta_2 \\ \vdots \\ \beta_B \\ \theta \end{pmatrix} \tag{14}$$

where $\beta_b = (\beta_{b1}, \dots, \beta_{bT})^T$ is the vector of time effects for batch b, and W_b is the corresponding design matrix for these time effects. X_b is the vector of treatment effect indicators for batch b. With Y_b, μ_b and V_b defined similarly, Equation 13 can be written as

$$\sum_{b=1}^{B} D_b^T V_b^{-1} (Y_b - \mu_b) = 0$$

where

$$D_b = \begin{pmatrix} 0 & \cdots & 0 & \frac{\partial \mu_b}{\partial \beta_b} & 0 & \cdots & 0 & \frac{\partial \mu_b}{\partial \theta} \end{pmatrix}.$$

Hence we can write the variance of the estimator β as

$$\left(\sum_{b=1}^{B} D_b^T V_b^{-1} D_b \right)^{-1} = \begin{pmatrix} \frac{\partial \mu_1}{\partial \beta_1}^T V_1^{-1} \frac{\partial \mu_1}{\partial \beta_1} & \cdots & & \frac{\partial \mu_1}{\partial \beta_1}^T V_1^{-1} \frac{\partial \mu_1}{\partial \theta} \\ \vdots & \ddots & \vdots & \\ & \cdots & \frac{\partial \mu_B}{\partial \beta_B}^T V_B^{-1} \frac{\partial \mu_B}{\partial \beta_B} & \frac{\partial \mu_B}{\partial \beta_B}^T V_B^{-1} \frac{\partial \mu_B}{\partial \theta} \\ \frac{\partial \mu_1}{\partial \beta_1}^T V_1^{-1} \frac{\partial \mu_1}{\partial \theta} & \cdots & \frac{\partial \mu_B}{\partial \beta_B}^T V_B^{-1} \frac{\partial \mu_B}{\partial \theta} & \sum_{b=1}^{B} \frac{\partial \mu_b}{\partial \theta}^T V_b^{-1} \frac{\partial \mu_b}{\partial \theta} \end{pmatrix}^{-1}. \tag{15}$$

Interest is in the variance of $\hat{\theta}$, which is given by the bottom right entry of this matrix:

$$\begin{aligned} var(\hat{\theta}) &= \left[\sum_{b=1}^{B} \left(\frac{\partial \mu_b}{\partial \theta}^T V_b^{-1} \frac{\partial \mu_b}{\partial \theta} - \frac{\partial \mu_b}{\partial \theta}^T V_b^{-1} \frac{\partial \mu_b}{\partial \beta_b} \left\{ \frac{\partial \mu_b}{\partial \beta_b}^T V_b^{-1} \frac{\partial \mu_b}{\partial \beta_b} \right\}^{-1} \frac{\partial \mu_b}{\partial \beta_b}^T V_b^{-1} \frac{\partial \mu_b}{\partial \theta} \right) \right]^{-1} \\ &= \left(\sum_{b=1}^{B} \frac{1}{var_b(\hat{\theta})} \right)^{-1} \end{aligned} \tag{16}$$

where $var_b(\hat{\theta})$ is the variance of the treatment effect estimator obtained when batch b is considered independently.

□

2 Code to replicate power calculations

This code uses version 1.6 of the `swdpwr` R package [1].

```
###################
# Demonstration for PACT-HF study #
###################
#2 batches of a 5-sequence, 6-period stepped wedge design
#54 patients in each cluster in each period
#Baseline prevalence of 28%
#ICC 0.01
#Reduction to 21%

library(swdpwr)

onebatch <- matrix(c(c(0,1,1,1,1,1),c(0,0,1,1,1,1), c(0,0,0,1,1,1),
                       c(0,0,0,0,1,1), c(0,0,0,0,0,1)),5,6,byrow=TRUE)

completedesign <- rbind(onebatch, onebatch)

PACTHF_K <- 54

# ICC=0.01, Hussey and Hughes within-cluster correlation structure
PACTHF_alpha0 <- 0.01
PACTHF_alpha1 <- 0.01

# P(outcome|control) = 0.28
# P(outcome|treatment) = 0.21

# First: assume that there is no underlying trend in probability of outcome
PACTHF_meanresponse_start = 0.28
PACTHF_meanresponse_end0 = 0.28
PACTHF_meanresponse_end1 = 0.21
PACTHFpower_nochange <- swdpower(K = PACTHF_K, design = completedesign,
                      family = "binomial", model = "marginal",
```

```
                        link = "logit", type = "cross-sectional",
                        meanresponse_start =  PACTHF_meanresponse_start,
                        meanresponse_end0 = PACTHF_meanresponse_end0,
                        meanresponse_end1 = PACTHF_meanresponse_end1,
                        typeIerror = 0.05, alpha0 = PACTHF_alpha0, alpha1 = PACTHF_alpha1)
#Power is 98.8%

#Allowing for baseline prevalence to change over time
PACTHF_batch1 <- swdpower(K = PACTHF_K, design = onebatch,
                               family = "binomial", model = "marginal",
                               link = "logit", type = "cross-sectional",
                               meanresponse_start =  0.30,
                               meanresponse_end0 = 0.29,
                               meanresponse_end1 = 0.2175,
                               typeIerror = 0.05, alpha0 = PACTHF_alpha0, alpha1 = PACTHF_alpha1)



PACTHF_batch2 <- swdpower(K = PACTHF_K, design = onebatch,
                             family = "binomial", model = "marginal",
                             link = "logit", type = "cross-sectional",
                             meanresponse_start =  0.29,
                             meanresponse_end0 = 0.28,
                             meanresponse_end1 = 0.21,
                             typeIerror = 0.05, alpha0 = PACTHF_alpha0, alpha1 = PACTHF_alpha1)

#Variance of treatment effect estimator for each batch:
PACTHFtreateff_batch1 <- abs(as.numeric(PACTHF_batch1$treatment.effect.beta))
PACTHFpower_batch1 <- as.numeric(PACTHF_batch1$Power)
PACTHFvar_batch1 <-  1/(( qnorm(1-PACTHFpower_batch1) + qnorm(0.025))/PACTHFtreateff_batch1)^2

PACTHFtreateff_batch2 <- abs(as.numeric(PACTHF_batch2$treatment.effect.beta]))
PACTHFpower_batch2 <- as.numeric(PACTHF_batch2$Power)
PACTHFvar_batch2 <-  1/(( qnorm(1-PACTHFpower_batch2) + qnorm(0.025))/PACTHFtreateff_batch2)^2
```

```
#The effect size we wish to detect is -0.38
PACTHF_var_batches <- 1/(1/PACTHFvar_batch1 + 1/PACTHFvar_batch2)
PACTHFpower_batches <- pnorm(qnorm(0.025)+0.38/sqrt(PACTHF_var_batches))
#Power is 80.7698%
```

References

[1] J. Chen, X. Zhou, F. Li, and D. Spiegelman. swdpwr: A SAS macro and an R package for power calculation in stepped wedge cluster randomized trials. *ArXiV*, page arxiv:2011.06031v1, 2020.

[2] S. L. Zeger and K.-Y. Liang. Longitudinal data analysis for discrete and continuous outcomes. *Biometrics*, 42:121–130, 1986.
